# Fast 3-T MR-guided transrectal prostate biopsy using an in-room tablet device for needle guide alignment: a feasibility study

**DOI:** 10.1007/s00330-018-5497-9

**Published:** 2018-05-22

**Authors:** Christiaan G. Overduin, Jan Heidkamp, Eva Rothgang, Jelle O. Barentsz, Frank de Lange, Jurgen J. Fütterer

**Affiliations:** 1 Department of Radiology and Nuclear Medicine, Radboud University Medical Center, P.O. Box 9101 (767), 6500 HB Nijmegen, The Netherlands; 2 Siemens Healthcare GmbH, Erlangen, Germany; 3 MIRA Institute for Biomedical Engineering and Technical Medicine, University of Twente, Enschede, The Netherlands

**Keywords:** Magnetic resonance imaging, Prostate cancer, Image-guided biopsy, Tablet computers, Operative time

## Abstract

**Objectives:**

To assess the feasibility of adding a tablet device inside the scanner room to assist needle-guide alignment during magnetic resonance (MR)-guided transrectal prostate biopsy.

**Methods:**

Twenty patients with one cancer-suspicious region (CSR) with PI-RADS score ≥ 4 on diagnostic multiparametric MRI were prospectively enrolled. Two orthogonal scan planes of an MR fluoroscopy sequence (~3 images/s) were aligned to the CSR and needle-guide pivoting point. Targeting was achieved by manipulating the needle-guide under MR fluoroscopy feedback on the in-room tablet device. Technical feasibility and targeting success were assessed. Complications and biopsy procedure times were also recorded.

**Results:**

Needle-guide alignment with the in-room tablet device was technically successful in all patients and allowed sampling after a single alignment step in 19/20 (95%) CSRs (median size 14 mm, range: 4-45). Biopsy cores contained cancer in 18/20 patients. There were no per-procedural or post-biopsy complications. Using the tablet device, the mean time to first biopsy was 5.8 ± 1.0 min and the mean total procedure time was 23.7 ± 4.1 min.

**Conclusions:**

Use of an in-room tablet device to assist needle-guide alignment was feasible and safe during MR-guided transrectal prostate biopsy. Initial experience indicates potential for procedure time reduction.

**Key Points:**

• *Performing MR-guided prostate biopsy using an in-room tablet device is feasible.*

• *CSRs could be sampled after a single alignment step in 19/20 patients.*

• *The mean procedure time for biopsy with the tablet device was 23.7 min.*

**Electronic supplementary material:**

The online version of this article (10.1007/s00330-018-5497-9) contains supplementary material, which is available to authorized users.

## Introduction

Prostate cancer (PCa) is the most common non-cutaneous cancer diagnosed in men in developed countries, with an estimated 180,000 new cases in the US in 2016 [1]. Transrectal ultrasound (TRUS)-guided biopsy is the current standard technique to detect PCa. However, this technique has limited sensitivity, and false-negative rates up to 32% have been reported for initial 6- to 12-core systematic biopsy [2, 3]. Repeated biopsy sessions have shown little impact on cancer detection [4, 5].

In recent years, multiparametric magnetic resonance (MR) imaging has evolved toward a mature imaging modality to detect PCa with high localization accuracy [6, 7]. Consequently, MR imaging (MRI) has been proposed in targeting biopsies toward cancer-suspicious regions (CSRs) [8], and several studies have demonstrated high diagnostic performance using in-bore MR-guided transrectal prostate biopsy in patients with initial negative TRUS biopsy [9–11]. Nevertheless, an important concern for in-bore biopsy is that the repeated needle guide adjustments requiring the physician to walk in and out of the MRI room are time consuming [12], and typical procedure times have been considerably longer than those of TRUS-guided biopsy [13].

To improve the current workflow, we propose a method for needle guide alignment using dedicated software integrated with MR fluoroscopy feedback, visualized on a tablet device inside the scanner room. This approach may eliminate the need for repeated needle guide repositioning and could potentially accelerate in-bore prostate biopsy procedures. Therefore, the purpose of this study was to assess the feasibility of adding a tablet device inside the MR room to assist needle guide alignment during 3-T MR-guided transrectal prostate biopsy.

## Materials and Methods

### Patient population

For this institutional review board (IRB)-approved feasibility study, 20 males scheduled for MR-guided prostate biopsy with a single CSR with a PI-RADS v2 [14] score ≥ 4 on diagnostic multiparametric prostate MRI (mpMRI) were prospectively enrolled between June and October 2016. Indications for diagnostic prostate mpMRI and subsequent biopsy were either elevated PSA (> 4.0 ng/ml) and clinical suspicion of primary PCa (*n* = 12) or follow-up in patients under active surveillance for minimal low-risk PCa (Gleason score ≤ 6) diagnosed on previous TRUS-biopsy (*n* = 8). None of the patients had received previous prostate treatment. Informed consent was obtained from all patients. A summary of patient and lesion characteristics is given in Table 1.

**Table 1:** Pre-biopsy patient and lesion characteristics (*n* = 20)

	Median or mean (range) or *n* (%)
Patient age (years)	63 (54–78)
PSA level (ng/ml)	14.9 (4.8–48.1)
Previous negative TRUS-guided biopsy sessions	2 (0–7)
Prostate volume (ml)	62.2 (20-159)
PSA density	0.30 (0.05–0.93)
Lesion localization	
Peripheral zone	14 (70)
Transition zone	6 (30)
Lesion suspicion score	
PI-RADS 4	10 (50)
PI-RADS 5	10 (50)
Lesion size (mm)	14 (4–45)
Lesion size distribution	
≤ 10 mm	4 (20)
> 10 mm	16 (80)

### MRI safety assessment

Prior to IRB application for this study, an MR safety assessment was conducted to determine safe operation conditions for the tablet device (iPad 2, Apple, Cupertino, CA, USA) in a 3-T MRI suite. The magnetically induced displacement force on the tablet device was determined according to American Society for Testing of Materials (ASTM) standard test method F2052-06 [15]. The tablet device was attached to a non-magnetic fixture using a nylon string such that it hung free in space. Using a protractor with 1° graduated markings, the deflection angle due to magnetic attraction of the tablet device was recorded at multiple locations in the scanner room and varying distances from the bore entry. Additional ASTM safety tests [16] were conducted to establish whether the tablet device or wireless network connection affected MR image quality.

### In-room tablet device setup

**Fig. 1:**
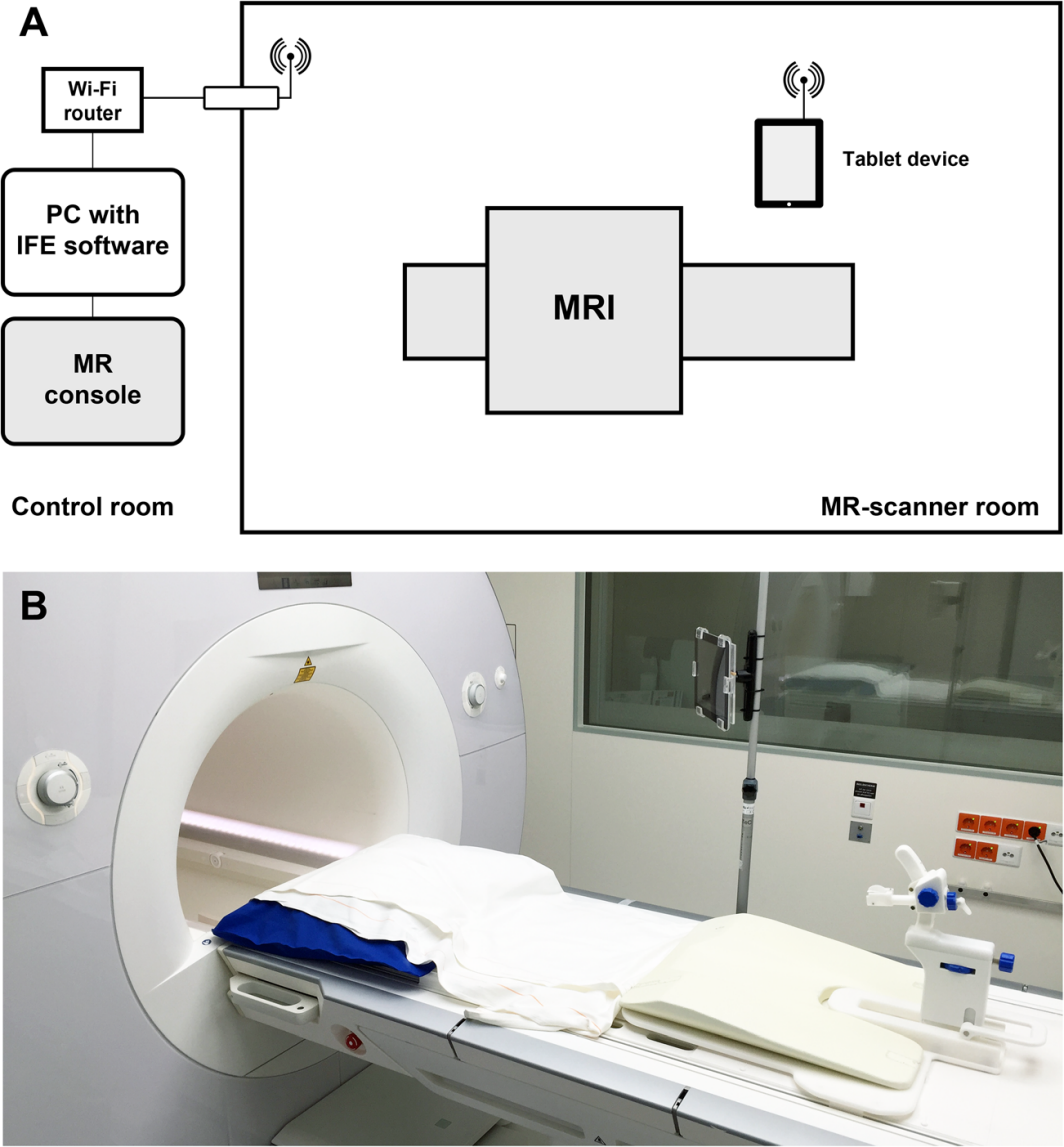
**a** Schematic diagram of the setup for prostate biopsy using an in-room tablet device. **b** Photograph shows the setup of the tablet device in the MR room

For the proposed method, the tablet device was installed in the MR room. The tablet device was connected to a stand-alone computer outside the scanner room via a remote desktop application (VNC Viewer, RealVNC, Cambridge, UK) on a secured wireless network connection (Fig. 1a). To prevent an accidental approach to the scanner bore during operation, the device was incorporated into a Perspex holder mounted to a non-magnetic fixture secured to the MR scanner table (Fig. 1b).

### MR-guided biopsy procedure

**Fig. 2:**
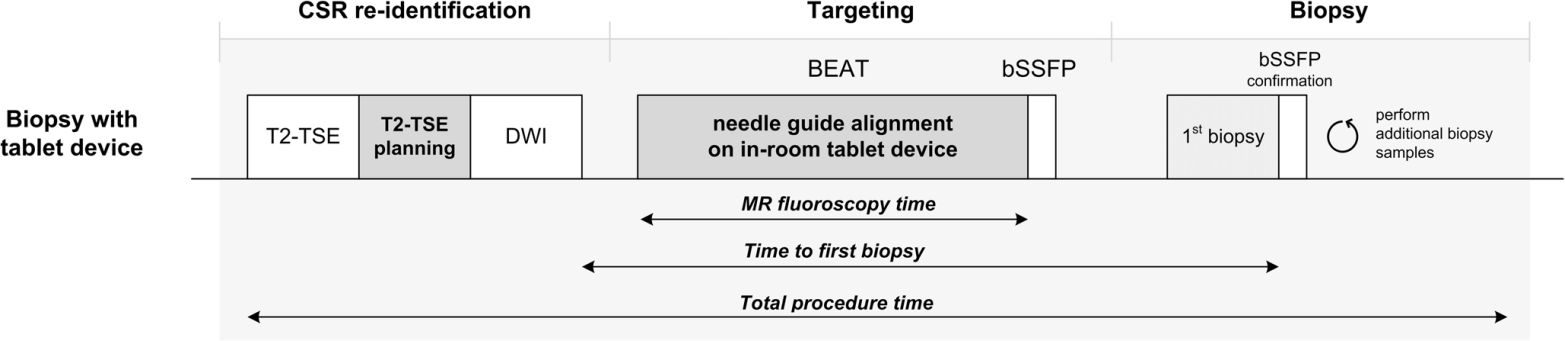
Schematic overview of procedure steps for biopsy using the in-room tablet device

All patients received antibiotic prophylaxis (oral ciprofloxacin, 2dd 500 mg) for 3 days, starting on the day before biopsy. All biopsy procedures were performed on a 3-T clinical MR system (MAGNETOM Skyra, Siemens, Erlangen, Germany) by one prostate interventionalist (C.O., 4-year experience). Patients were positioned in a prone position on the MR table. A commercially available transrectal biopsy device (DynaTrim, Invivo, Gainesville, FL, US) was used. Axial T2-weighted turbo spin echo (T2-TSE) and diffusion-weighted imaging (DWI) were acquired to reproduce pre-biopsy MRI findings. An additional extended axial T2-TSE data set incorporating the entire needle guide was acquired for planning purposes. On the stand-alone computer, two points were identified in the extended T2-TSE set using interventional planning software [Interactive Front End (IFE), Siemens Healthcare GmbH, Erlangen, Germany]: (1) the biopsy target, defined as the center of the lesion, and (2) the needle guide pivoting point, which had been previously determined at 9.2 cm from the proximal edge of the contrast-filled compartment of the needle guide in in-house experiments. The software then calculates the planned trajectory between these two points, representing the desired needle guide position to target the CSR (Supplemental Figure 1). Two orthogonal MR scan planes were aligned to this trajectory in axial and sagittal orientation and returned to the scanner console, providing the slice positions for an interactive real-time balanced steady-state free precession (BEAT) MR fluoroscopy sequence (temporal resolution ~3 imagings/s). The interventionalist then entered the MR room and manipulated the needle guide into both scan planes under MR fluoroscopy feedback visualized on the in-room tablet device while the patient was inside the scanner bore (Supplemental Video 1). Upon completion, short balanced steady-state free precession (bSSFP) scans were performed in axial and sagittal orientation to confirm correct alignment with the CSR. In case of correct alignment, subsequent biopsy was performed using an 18-gauge fully automatic MR-compatible biopsy gun (Invivo, Gainesville, FL, US). In case of incorrect alignment, additional routine manual adjustment steps were performed for correction. Axial and sagittal bSSFP confirmation scans were obtained to map each biopsy location. Detailed imaging parameters are summarized in Table 2. An overview of the biopsy procedure is shown in Fig. 2.

**Table 2:** Imaging protocol for MR-guided prostate biopsy with in-room tablet device

	Repetition time (ms)	Echo time (ms)	Flip angle (°)	No. of sections	Section thickness (mm)	Field of view (mm^2^)	Matrix size	Image orientation	Acq. time (min)	Additional parameters
T2-TSE	5850	87	160	19	3.0	192 × 192	256 × 256	Axial	3:07	Averages = 3
T2-TSE for trajectory planning	12540	87	160	45	3.0	192 × 192	256 × 256	Axial	1:40	Averages = 1
DWI	3300	63	90	19	3.0	240 × 256	120 × 128	Axial	4:49	b-values = 50, 400, 800 s/mm^2^
Interactive real-time balanced steady-state free precession (BEAT)	4.16	2.03	30	1	5.0	300 × 300	160 × 160	Axial; sagittal	-	Temporal resolution = 3 images/s
Balanced steady-state free precession (bSSFP)	4.56	2.28	65	5	3.0	280 × 280	256 × 256	Axial; sagittal	0:09

Per- and post-procedural complications were recorded with rates and description of adverse events. Complications were scored according to the Clavien grading system [17].

### Targeting success and procedure times

Technical feasibility and single-step targeting success of needle guide alignment were assessed. Single-step targeting success was defined as obtainment of a representative biopsy core directly after a single alignment step under MR fluoroscopy feedback on the in-room tablet device without requiring further needle guide repositioning. Representativity of each biopsy core was indicated on a five-point scale (i.e., 1 = not representative to 5 = representative) by a prostate radiologist reviewing each biopsy confirmation scan, where a score of ≥ 4 was considered a representative sample.

Biopsy procedure times were also recorded. The time to first biopsy was defined as the time from acquisition of the DWI sequence until the confirmation scan of the first biopsy of the CSR (Fig. 2). The total procedure time was defined as the time between localizer acquisition and the last scan of the biopsy procedure. Finally, the MR fluoroscopy time was defined as the duration the physician required to align the needle guide under MR fluoroscopy feedback. The setup time of the required hardware was not recorded as multiple subsequent biopsy procedures only required a single installation of ± 5-10 min.

### Histopathological analysis

After the biopsy procedures, core samples underwent histopathological workup with hematoxylin-eosin staining and were subsequently evaluated for the presence of PCa or benign findings. When applicable, a Gleason score was assigned.

## Results

### MRI safety

In the 3-T suite, it was found that the specified unfixed tablet device could be safely operated at ≥ 50 cm from the bore entry without significant magnetic attraction according to ASTM tolerance levels. No noticeable interferences or artifacts affecting MR image quality were observed because of the presence of the tablet device or wireless network connection.

### Biopsy procedures

A total of 20 CSRs was successfully sampled in 20 patients (1 per patient). A median of two biopsy cores (range: 2-3) was taken per CSR resulting in 46 biopsy samples. There were no per-procedural or post-biopsy complications.

**Fig. 3:**
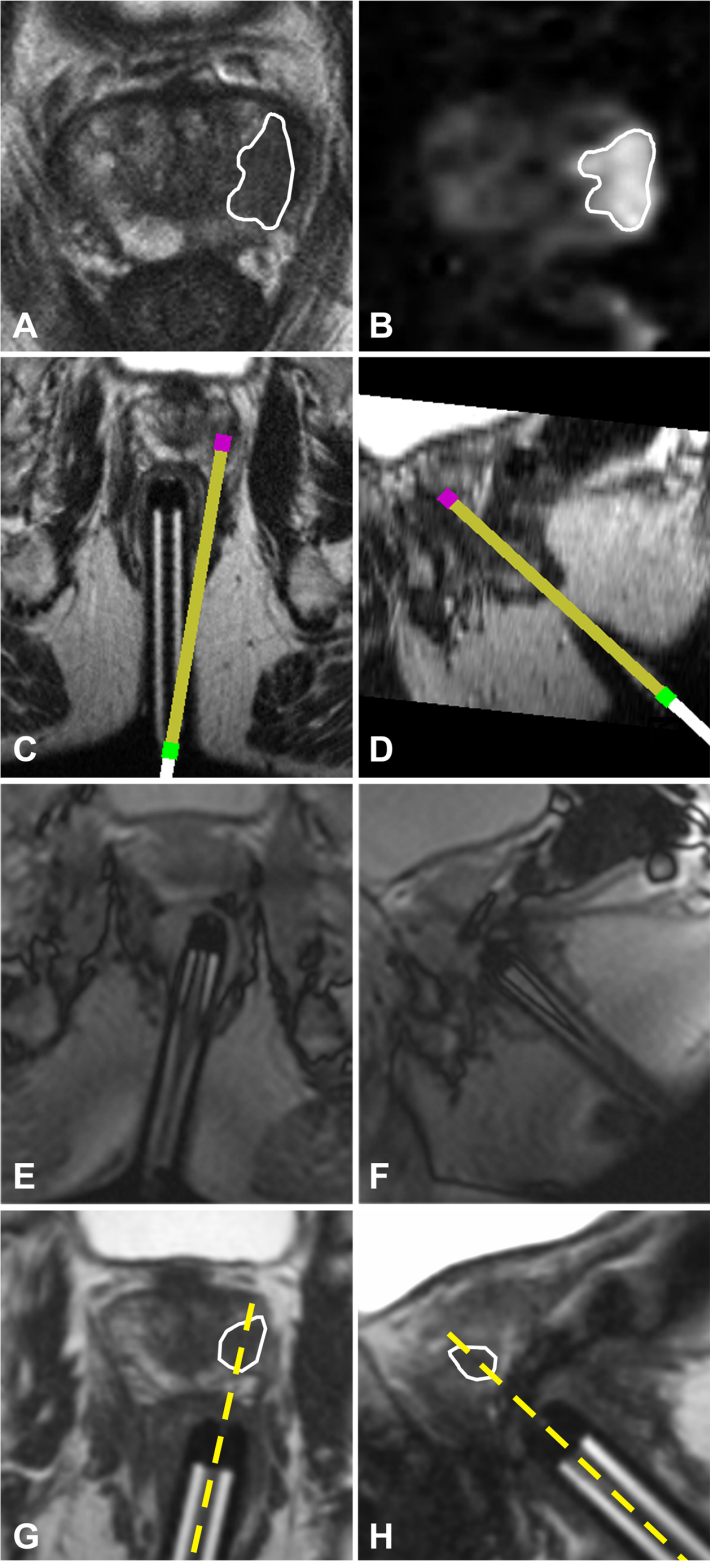
Images in a 63-year-old male with elevated PSA. (**a**) Axial diagnostic T2-weighted and (**b**) DW imaging (calculated high b-value image; 1400 s/mm^2^) shows a focal hypointense lesion with diffusion restriction (white outline) suspicious for PCa in the left peripheral zone. (**c**) Axial and (**d**) sagittal view of the needle guide trajectory planning. (**e**) Axial and (**f**) sagittal BEAT images aligned to the planned trajectory show the final needle guide position after alignment under MR fluoroscopy feedback displayed on the in-room tablet device. (**g**) Axial and (**h**) sagittal fast bSSFP scans confirm correct alignment of the needle guide to the CSR in both orientations (yellow dotted lines). Subsequent biopsy showed a Gleason 3 + 4 = 7 prostate cancer

Needle guide alignment using the in-room tablet device was technically feasible in all patients (Fig. 3). Single-step targeting was successful in all but one patient (19/20 lesions; 95%). In the latter patient, an additional routine repositioning step was needed because the lesion was located anteriorly outside of the maximum range of the biopsy device. After forward adjustment of the needle guide, biopsy was performed successfully. The median radiological score of biopsy representativity was 5 out of 5 (range: 4-5).

### Procedure times

**Fig. 4:**
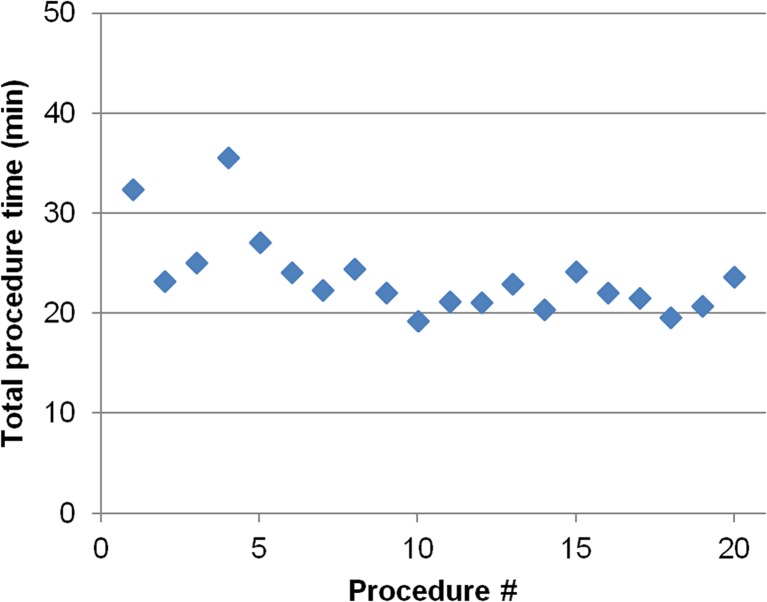
Total procedure times as function of procedure number

With use of the in-room tablet device, mean time to first biopsy was 5.8 ± 1.0 min and mean total procedure time was 23.7 ± 4.1 min. There was a downward trend in total procedure time with increasing procedure number, with the last ten procedures being performed in less than 25 min (Fig. 4). The mean MR fluoroscopy time was 1.1 ± 0.3 min.

### Histopathologic findings

Histopathology revealed prostate cancer in 18/20 (90%) patients. Gleason scores were 3+3 (3), 3+4 (10), 3+5 (1), 4+3 (2), 4+4 (1) and 4+5 (1). In total, 42 of 46 (91%) biopsy samples contained cancer. In two patients (10%), biopsy samples contained prostatitis.

## Discussion

This work described a method for needle guide alignment using an in-room tablet device during MR-guided transrectal prostate biopsy and demonstrated its feasibility in patients. Biopsy samples could be successfully obtained from each CSR after a single alignment step using the in-room tablet device in all but one patient.

In an effort to reduce procedure times of in-bore MR-guided prostate biopsy, alternative solutions have previously been proposed. Several groups have developed MR-compatible robotics to aid in in-bore prostate biopsy [18–20]. Another report has described an algorithm for automated phase-only cross correlation (POCC)-based needle-guide tracking [21]. Also, an MR-compatible display screen could be used instead of the proposed tablet device to visualize MR fluoroscopy images inside the MR room. An advantage however of the method proposed here is its relative simplicity, requiring minimal additional equipment costs (i.e., a tablet device and wireless network connection).

Disadvantages of the tablet device are that the screen is relatively small and MRI safety needs to be concerned. MRI safety assessment showed that the tablet device could be safely operated in a 3-T MRI suite at distances ≥ 50 cm from the bore entry. This is in line with previous results in a 1.5-T MR environment [22]. Nevertheless, when a tablet device is applied for different applications and MRI suites caution should be taken and device safety assessed in each individual setting by local MR safety authorities.

The mean total procedure time for biopsy using the in-room approach was 23.7 min, with the last ten procedures all performed in < 25 min including ~2 min additional scanning time required to obtain the extended T2-TSE data set for trajectory planning. Compared with the median procedure times of transrectal in-bore MR-guided prostate biopsy reported in the literature (30-68 min) [13], this presents a considerable time improvement. Another study described initial results using a robotic manipulator and reported biopsy of one CSR per patient in a median of 37 min (range: 23-61) [23]. Another study used a needle-guide tracking sequence enabling biopsy of a median of two CSRs (range: 1-4) per patient in a median of 32 min (range: 14-48), without diagnostic scans at the beginning of the biopsy procedure. In our preliminary cohort, needle guide alignment using the in-room tablet device allowed biopsy of the CSR after a single adjustment step in almost all patients. With the manipulation under MR fluoroscopy being performed in ~1 min and the physician only needing to walk between the MR and control room once, this is the main area where the time improvement was achieved in this study.

Some aspects of the in-room targeting process may still be optimized. Scanning time could be saved by incorporating a T2-TSE sequence that can be used for CSR-reidentification and planning of the needle guide trajectory. Also, optimization of the planning software or integration with the scanner platform could improve the slice positioning workflow and allow further acceleration of the biopsy procedure.

One issue with the presented method is that MR fluoroscopy guidance comes with the prerequisite of manipulating the needle guide in the center of the bore while the patient is on the scanner table. Potentially, some performing physicians may be unable to reach the needle guide inside the magnet. Also, the in-room method is presently only compatible with transrectal biopsy using a specific commercial biopsy device in combination with interventional planning software of one vendor.

The most important limitations to this feasibility study are the relatively small population and investigation of biopsy of only one CSR per patient with a PI-RADS score ≥ 4. Finally, we acknowledge that the future of in-bore biopsy is unclear as recently interest in MRI-TRUS fusion biopsy has been increasing as a targeted biopsy alternative. Some initial studies have shown promising results using fusion biopsy [24, 25]. One disadvantage is that MRI-TRUS fusion systems often require pre-segmentation of tumors on the diagnostic MRI, which can also be a time-consuming process. Ultimately, validation in larger cohorts is needed to determine which lesions are best amenable to each biopsy technique. In cases of discrepancies between imaging and fusion biopsy findings, an important role could remain for in-bore biopsy. Moreover, physician preference of technique and site experience may also play a part.

In conclusion, use of an in-room tablet device to assist needle guide alignment was feasible and safe during 3-T MR-guided transrectal prostate biopsy. Our initial clinical experience indicates potential for procedure time reduction, which could be of value to increase the clinical applicability of this biopsy technique.

## Electronic supplementary material

Supplemental Figure 1 (DOCX 5815 kb)

Supplemental Video 1 (MP4 2543 kb)

## Abbreviations

ASTM: American Society for Testing of Materials
bSSFP: Balanced steady-state free precession
CSR: Cancer-suspicious region
DWI: Diffusion-weighted imaging
IFE: Interactive front end
mpMRI: Multiparametric MRI
MRI: Magnetic resonance imaging
PCa: Prostate cancer
PI-RADS: Prostate Imaging Reporting Archiving and Data System
PSA: Prostate-specific antigen
TRUS: Transrectal ultrasound
TSE: Turbo spin echo
