# Supplementary material for: Fast 3-T MR-guided transrectal prostate biopsy using an in-room tablet device for needle guide alignment: a feasibility study
Source: Eur Radiol. 2018 May 22;28(11):4824–31. doi: 10.1007/s00330-018-5497-9 (PMC6182740; doi:10.1007/s00330-018-5497-9)
Supplement: Supplementary file 1 — (DOCX 5815 kb) [file 330_2018_5497_MOESM1_ESM.docx]

**Supplementary material**

**
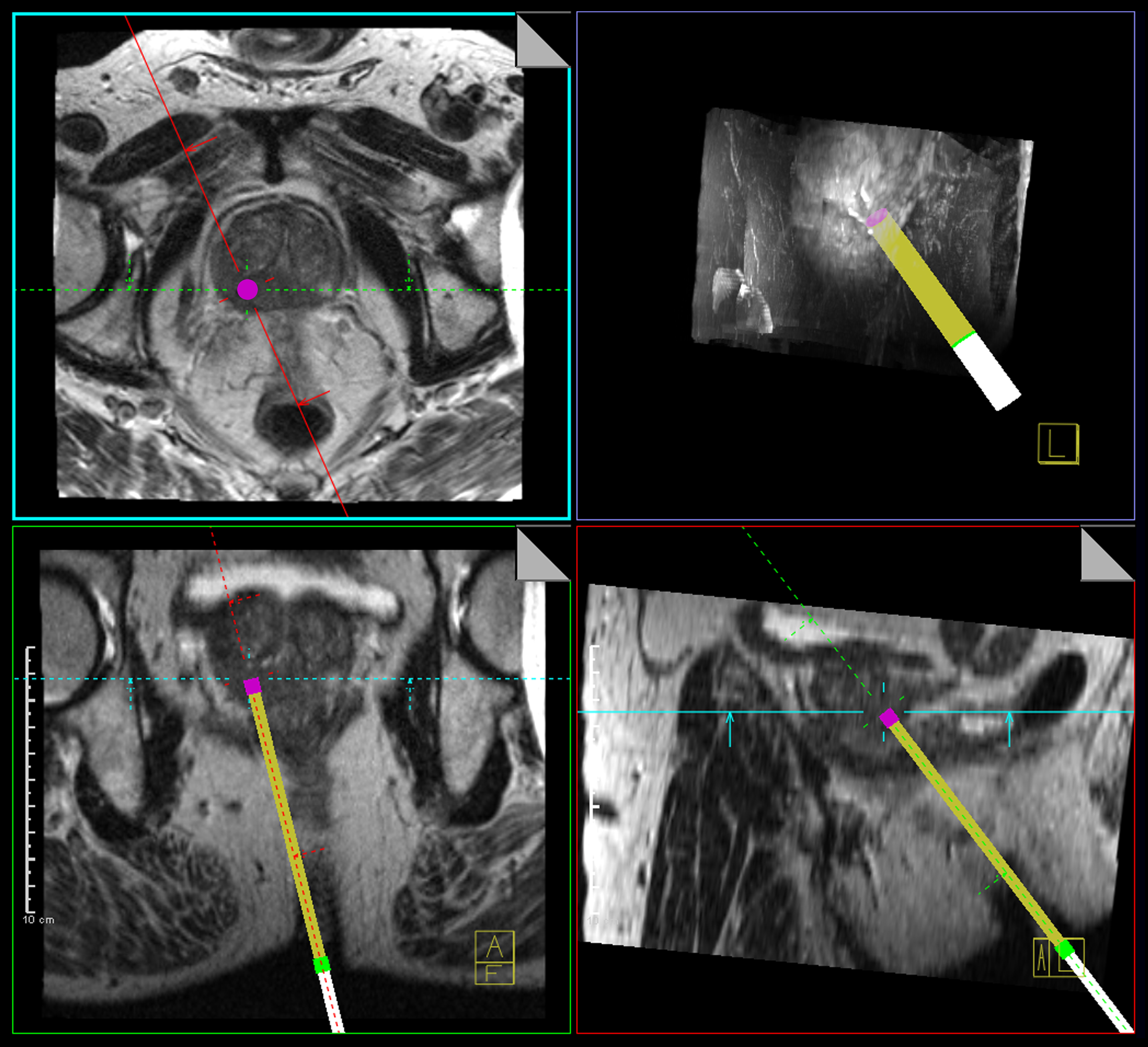
Supplemental Figure 1** – Overview of the IFE planning module shows a multiplanar reconstruction of the T2-TSE planning image set. The biopsy target (pink) and the needle guide pivoting point (green) are indicated, as well as the planned trajectory through these

points (yellow path) representing the desired needle guide trajectory to target the CSR.

**Supplemental Video 1** – Screen recording of the image displayed on the in-room tablet device during needle guide manipulation in one of the biopsy procedures. MR fluoroscopy images are visualized within the IFE software with the previously obtained planned needle guide trajectory overlaid. Using the touch screen of the tablet device, the interventionalist can switch between views of the different MR slices.
